# Corrosion behaviour of micro-arc oxidation coatings on Mg–2Sr prepared in poly(ethylene glycol)-incorporated electrolytes

**DOI:** 10.1039/c7ra12497j

**Published:** 2018-01-22

**Authors:** Dandan Gao, Jinhe Dou, Cheng Hu, Huijun Yu, Chuanzhong Chen

**Affiliations:** Shenzhen Research Institute of Shandong University Shenzhen 518057 Guangdong P. R. China yhj2001@sdu.edu.cn czchen@sdu.edu.cn +86 531 88395991 +86 531 88395991; Key Laboratory of High-efficiency and Clean Mechanical Manufacture (Shandong University), Ministry of Education, School of Mechanical Engineering, Shandong University Ji'nan 250061 Shandong P. R. China yhj2001@sdu.edu.cn; National Demonstration Center for Experimental Mechanical Engineering Education (Shandong University), School of Mechanical Engineering, Shandong University Ji'nan 250061 Shandong P. R. China; Key Laboratory for Liquid-Solid Structural Evolution and Processing of Materials, (Ministry of Education), School of Materials Science and Engineering, Shandong University Ji'nan 250061 Shandong P. R. China czchen@sdu.edu.cn

## Abstract

Microarc oxidized calcium phosphate (CaP) ceramic coatings were fabricated on Mg–2Sr alloy from silicate electrolytes with different concentration gradient poly(ethylene glycol) (PEG_1000_). The microstructure, phase and degradability of the ceramic coatings were evaluated by scanning electron microscopy (SEM), X-ray diffraction (XRD) and simulation body fluid (SBF) immersion tests respectively. An electrochemical workstation was used to investigate the electrochemical corrosion properties of the coatings. It is found that microstructure, thickness, adhesive strength and degradation rate are influenced by PEG_1000_ incorporation through adjusting the electrolyte activity and then altering the coating growth mechanism. Similar thicknesses (39.0–42.2 μm) are observed in PEG_1000_-containing coatings while their PEG_1000_-free counterparts possess the maximum value (51.5 μm). The weight gain in the first two days of SBF immersion suggests that a new layer containing CaP apatites is generated. Results show that ceramic coatings prepared in the electrolyte containing 8 g L^−1^ PEG_1000_ exhibits the highest corrosion resistance and lowest degradation rate.

## Abstract 2

The highest corrosion resistance and lowest biodegradation are observed on the ceramic coating prepared in electrolytes containing 8 g L^−1^ PEG_1000_.

## Graphical abstract

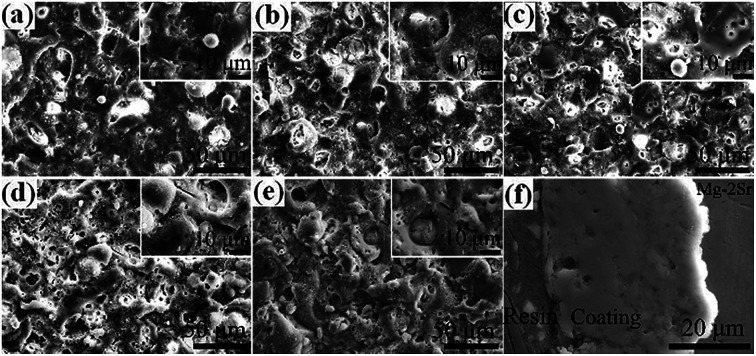

## 1. Introduction

Magnesium (Mg) based alloys show exciting potentials in biocompatible, osteoconductive, degradable implants ascribed to their excellent mechanical properties, intriguing biocompatibility and spontaneous biodegradability.^1–3^ Concretely, the elastic modulus and compressive yield strength of Mg are closer to those of natural bone, which is beneficial to weaken stress shielding effects.^4,5^ Moreover, as the 4th most abundant cation in the human body, approximately half of the Mg is found in bone tissues for its essential role in human metabolism.^6,7^ Besides, corrosion products *in vivo* can be eliminated easily by the body without any adverse side effects.^8^ Belonging to group II in the periodic table, Sr is a promising plant growth stimulator similar to Ca in the same column.^9,10^ It is feasible to choose Sr-incorporated Mg based alloys to serve as bone implants. Hydroxyapatite (HA) bioactive cement incorporated with Sr (Ca_10−*x*_Sr_*x*_(PO_4_)_6_(OH)_2_, Sr-HA) have been found to be favorable for bone repair.^10–13^ Sr also facilitates cell growth, proliferation and healing around the bone implants thus enhancing osteoblastic activity and bone formation *in vivo*.^14,15^ Additionally, 2% of alloying Sr brings Mg based alloys improved mechanical properties and reduced corrosion rates.^16,17^ Altogether, Mg–2Sr is an attractive candidate for biocompatible Mg based alloys implants.

However, rapid corrosion leads to structural failure of implants before completing healing of host tissues.^1,18–20^ The aggregation of corrosion products such as hydrogen gas results in the formation of gas bubbles, which prolongs the healing process of bone tissues.^21^ The induced change of the physical environment disturbs implants' stability and mechanical integrity before the end of their service periods.

The reduced corrosion rate and local gas cavity may depend on the followings: purity, alloying and surface modification.^2,22,23^ Implants equipped with both sufficient mechanical properties and appropriate corrosion rates are expected *via* alloying firstly and coating subsequently. Hence, it is essential to seek an effective surface modification technology to provide a relatively uniform, dense, anti-corrosive and even bioactive coating on the selected Mg based alloy. Investigations^24,25^ have manifested that micro-arc oxidation (MAO) is a particularly prospective surface modification technology available for substrate strengthening and biocompatibility enhancing concerning electroplating,^26^ chemical conversion method,^27^ biomimetic approach,^28^ electrochemical deposition,^29,30^ anodizing^31^*etc.* Particularly, MAO is well-known for its capacity of generating *in situ* grown porous and homogeneous oxide coatings metallurgically bond to the substrate with strong adhesion.^32^ MAO process can be controlled easily at room temperature and repeated on the substrate for multiple times.^33^ It requires less energy consumption at lower reaction temperature in alkalescent electrolytes, providing an environmental-friendly solution for surface treatment.^34^

The phases, microstructures, corrosion resistance, adhesion and degradation of MAO coatings are determined by substrate designing, electrical parameters and electrolyte recipe adjusting, respectively.^22,35,36^ During the process, electrolyte components could diffuse into the formed coating and then influence the coating growth mechanism.^37^ Given that the electrolyte recipe plays a vital role in determining the final performance of coatings. Additives incorporated into the electrolyte are beneficial to the MAO process by optimising microstructures of ceramic coatings to some extent. Micro-molecule additives including KF, NH_4_HF_2_, C_3_H_8_O_3_ and H_2_O_2_ have been studied on AZ91D and ZK60 alloys^38,39^ widely. However, macro-molecule additives such as poly(ethylene glycol) (PEG_1000_) are rarely investigated. PEG is water soluble and nontoxic, which is a potential green candidate for additives in electrolyte system. It has been applied widely in pharmaceutical, textile, cosmetic industry as lubricant, dispersant and softener. Besides, it is one of effective nonionic surfactants especially for reactions of the interface with functional –CH_2_–CH_2_–O– segments, which can accelerate or optimise reaction processes.^40,41^ In this study, PEG_1000_ additives are added into silicate electrolytes with gradient concentrations of 0 g L^−1^, 4 g L^−1^, 8 g L^−1^, 12 g L^−1^, 16 g L^−1^, which are referred as P0, P1, P2, P3 and P4, respectively in the following sections. The effects of PEG_1000_ on the performance of MAO coatings on Mg–2Sr surface are studied and the optimal concentration is also summarized.

## 2. Materials and methods

### 2.1. Substrate and coating preparation

Mg–2Sr was cast using commercial purity Mg (99.99%) and Mg–21Sr (99.99%) master alloy in an electronic resistance furnace under a protective atmosphere (SF_6_ : CO_2_ = 1 : 200). Raw materials and tools were preheated to 250 °C. Mg ingot was firstly melted at 700 °C. Mg–21Sr master alloys with the calculated quantity were then added at 710 °C along with a subsequent holding period of 20 min to ensure sufficient fusion. The dissolved melt was stirred uniformly before being poured into the preheated graphite mold. Lastly, Mg–2Sr alloy was cut into rectangular specimens with the size of 8 × 10 × 10 mm^3^ after homogenized at 400 °C for 16 h, which were mechanically polished with carborundum waterproof abrasive paper up to 1000 grit, degreased with acetone following by rinsing with distilled water and absolute ethyl alcohol, respectively before oxidation.

The electrolyte recipes containing calcium, phosphorus and silicon were prepared from the base electrolyte (15 g L^−1^ NaSiO_3_, 5 g L^−1^ KOH) and CaHPO_4_ (10 g L^−1^), NH_4_HF_2_ (7 g L^−1^), C_3_H_8_O_3_ (5 ml L^−1^), H_2_O_2_ (5 ml L^−1^) while PEG_1000_ of gradient concentrations aforementioned are incorporated in the end. All solutions were made from analytical grade reagents and distilled water. During the oxidation, the cleaned and dried specimens acted as an anode while the stainless steel container served as a cathode. The electrolyte temperature was controlled to be stable at 35 °C or lower by a modified cooling water circulating pump. MAO process was controlled to 15 min and electrical parameters are listed in detail (Table 1). Finally, MAO-coated samples were dried with a blower after rinsed in distilled water and absolute ethyl alcohol.

**Table 1:** Electrical parameters for MAO process

Positive voltage (V)	Frequency (Hz)	Negative voltage (V)	Positive duty ratio (%)	Negative duty ratio (%)	Ratio of positive and negative pulse
450	550	40	30	20	1 : 1

### 2.2. Coating characterization

Phase constitutions were studied by X-ray diffractometer (Dmax-2500, Rigaku) with Cu-Kα radiation at a scanning speed of 2° min^−1^ under the voltage of 40 kV and the current of 30 mA. Fourier transform infrared (FTIR) spectroscopy (Tensor-37, BRUKER, Germany) was used to analyze the functional groups with the resolution of 4 cm^−1^ and scanning period of 16 s. Scanning electron microscope (SEM, JSM-6380LA, Japan) and energy dispersive X-ray spectrometry (EDX, JED-2300, Japan) were performed to observe surface and cross-section morphologies as well as the compositions of coatings before and after degradation tests in SBF. MINITEST 600B FN2 microprocessor coating thickness gauge (Elektro-physik Koln, Germany) was utilized to measure the thicknesses of MAO coatings and the final values reported were the average of nine replicate measurements. Scratch test was carried out on a scratch tester (WS-2005, Lanzhou Institute of Chemical Physics, Chinese Academy of Sciences, Lanzhou, China). A maximum 20 N load was applied at a loading speed of 20 N min^−1^ at the speed of 3 mm min^−1^.

### 2.3. Degradation and bioactivity assessment

MAO treated and untreated samples were immersed in SBF solution with ion concentrations almost equal to those in human blood plasma.^42^ The 1.0 SBF was prepared by dissolving the reagents of NaCl, NaHCO_3_, KCl, K_2_HPO_4_·3H_2_O, MgCl_2_·6H_2_O, 1.0 mol L^−1^ HCl, CaCl_2_ and Na_2_SO_4_ in distilled water at 36.5 ± 0.5 °C one by one according to the order above. Then pH was adjusted to 7.2–7.4 with trishydroxymethylaminomethane ((CH_2_OH)_3_CNH_2_) and hydrochloric acid at 36.5 °C.^42^ Each sample with 440 mm^2^ immersion area was soaked in a 60 ml plastic vial filled with 40 ml of SBF solution and placed in the thermostat water bath at 36.5 °C. The ratio of sample's surface area to SBF volume was 110 mm^2^ : 10 ml and SBF solution was renewed every 2 days and kept colorless, stable without deposit during immersion for 1 day, 2 days, 7 days, 14 days, 21 days and 28 days. Samples were removed from the SBF and washed with distilled water after any immersion period.

### 2.4. Electrochemical corrosion behaviour

Potentiodynamic polarization tests were carried out on the substrate and MAO-coated samples by the electrochemical workstation (Princeton PARSTAT 2273, USA) in a 200 ml electrolyser with a typical three-electrode setup. Saturated calomel electrode (SCE) was used as the reference electrode while a platinum sheet acted as the counter electrode. The working electrode was wrapped with paraffin film leaving a 1.0 cm^2^ area in contact with corrosive SBF solution. The temperature was maintained at (36.5 ± 0.5) °C. The potential was scanned from −2500 mV to 0 V at a rate of 5 mV s^−1^ when the corrosion potential remained stable.

## 3. Results

### 3.1. Phase analysis

#### 3.1.1. XRD analysis

**Fig. 1:**
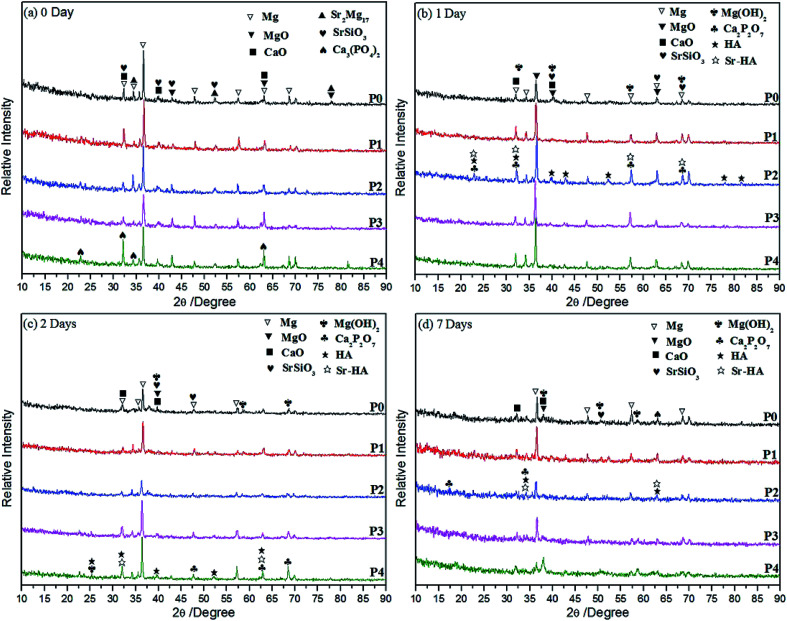
XRD patterns of MAO coatings formed in different electrolytes before (a) and after (b) 1 day, (c) 2 days, (d) 7 days immersion in SBF solutions.

XRD patterns of the MAO coatings (P0 to P4) prepared in silicate electrolytes with different levels of PEG_1000_ before and after SBF soaking are shown in Fig. 1. The MAO coatings are mainly composed of Mg, MgO, CaO, SrSiO_3_, Sr_2_Mg_17_ and Ca_3_(PO_4_)_2_ (tricalcium phosphate, TCP) regardless of the different PEG_1000_ concentrations. (Fig. 1(a)). In addition, the intensities of the magnesium phases (Mg, Sr_2_Mg_17_) are relatively stronger for the Mg-based substrate meaning higher crystallinity. It can also be found that the intensities of MgO and TCP are directly related to the increasing PEG_1000_ concentration. The presence of SrSiO_3_ and Ca_3_(PO_4_)_2_ indicates that elements from substrate (Sr) and electrolyte (Ca, P and Si) have combined and generated new composites in MAO coatings successfully. Particularly, TCP is a resorbable temporary bone space filler material with high solubility in human body, which can also stimulate the growth of new bone tissue along with the repairing process.^43^ As expected, the TCP will be transformed to HA under the function of body fluids after implantation. Similar results *in vitro* immersion are observed in Fig. 1(b)–(d) for 1 day, 2 days and 7 days. Formation of new phases including Ca_2_P_2_O_7_ (CCP), Mg(OH)_2_, HA and Sr-HA, is discovered after soaking for 1 day demonstrated in Fig. 1(b)–(d) while the pre-existing phases can still be picked up except for the soluble TCP phase. CCP is another desired biocompatible phase acting as the precursor of HA while Mg(OH)_2_ is one of the corrosion products beneficial to the formation and growth of HA.^1^ As the main mineral constituent of teeth and bones, HA is of excellent biocompatibility to hard tissues. Additionally, it can directly bond to the bone without toxicity and is considered as one of the most suitable ceramic materials for hard tissues replacement implants.^1,44^ Ca_10−*x*_Sr_*x*_(PO_4_)_6_(OH)_2_) (Sr-HA) is also known to enhance cell growth and proliferation and healing around bone implants, in which element Sr partially replaces the position of element Ca.^45^ Besides, the relative intensities of HA and Sr-HA detected in coating P2 are higher than those of the other four, implying better biocompatibility and bioactivity of the coating prepared in electrolyte system with 8 g L^−1^ PEG_1000_. Much weaker intensities corresponding to the substrate (Mg) after 2 days soaking in the SBF (Fig. 1(c)) can be attributed to a thicker layer harder to be penetrated has been formed on surfaces of MAO-coated samples. With the prolonged exposure period for 7 days, the intensities of the five coatings are lowered with regard to the same phases shown in Fig. 1(b) and (c). This may be because some amorphous phases have been generated.

#### 3.1.2. FT-IR analysis

**Fig. 2:**
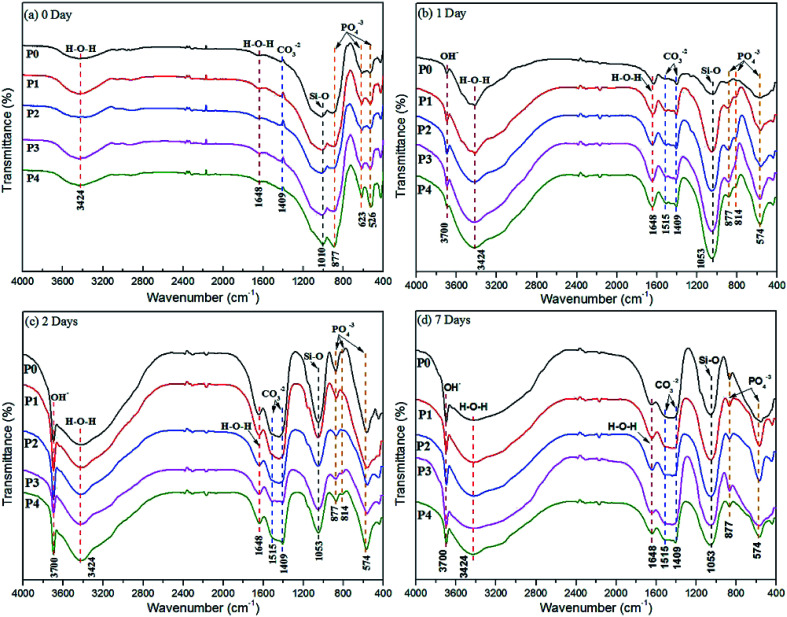
FT-IR spectra of MAO coatings formed in different electrolytes before (a) and after (b) 1 day, (c) 2 days, and (d) 7 days immersion in SBF solutions.

The FT-IR spectra of MAO-coated samples before and after vitro immersion in SBF for 1 day, 2 days and 7 days are compared in Fig. 2, where extra information about the amorphous phases and chemical compositions of the coatings can be obtained. Obvious differences between the as prepared and soaked coatings are found throughout the four images. Bands of certain chemical compositions are weaker in Fig. 2(a) than those of in other ones. No characteristic frequency of the O–H stretching vibration at 3700 cm^−1^ is observed in Fig. 2(a), implying that Mg(OH)_2_ is free in the prepared coatings.^46^ Weak, broad bands at 3424 cm^−1^ and 1648 cm^−1^ are attributed to stretching absorption band of free water and bending band of crystal water in Fig. 2(a) while these become stronger and sharper after SBF soaking.^47^ Split bands at 1515 cm^−1^ and 1409 cm^−1^ in Fig. 2(b)–(d) are due to the V_3_ anti-symmetric stretching mode of carbonate (CO_3_^2−^) groups.^48^ However, a feeble band at 1409 cm^−1^ corresponding to CO_3_^2−^ presents in Fig. 2(a) only. The weak band at 1010 cm^−1^ in Fig. 2(a) and wide absorption band at 1053 cm^−1^ in remaining three ones are assigned to the Si–O (s, asym) asymmetric stretching mode.^49^ Bands at 877 cm^−1^, 814 cm^−1^, 574 cm^−1^ in Fig. 2(b)–(d) as well as 623 cm^−1^, 526 cm^−1^ in Fig. 2(a) are attributed to PO_4_^3−^, HPO_4_^2−^ and (or) P_2_O_7_^4−^ groups.^50^ In addition, bands below 500 cm^−1^ are ascribed to the presence of M–O and (or) M–F (M = Mg, Ca) and (or) lattice vibrations.^51^ Moreover, tendencies of spectra are similar to each other for different immersion periods as seen in Fig. 2(b)–(d). The main difference amongst Fig. 2(b)–(d) is the absence of the band at 814 cm^−1^ in Fig. 2(d), demonstrating the removal of HPO_4_^2−^. The formation of new functional groups in the coating after SBF soaking is confirmed during the FTIR analysis.

### 3.2. Microstructure

#### 3.2.1. Surface and cross-section morphology before SBF immersion

**Fig. 3:**
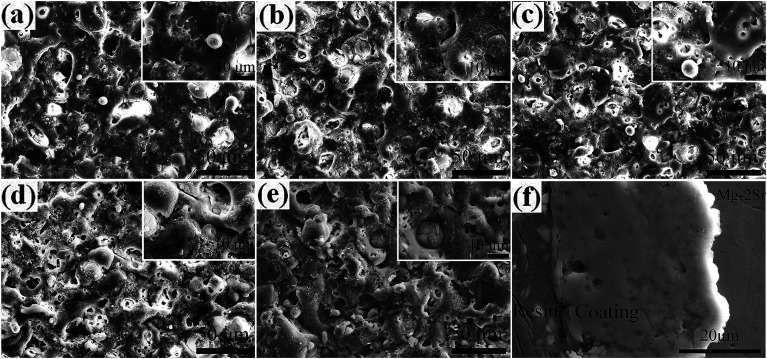
Surface morphologies of the MAO coatings formed in silicate electrolyte system with (a) 0 g L^−1^, (b) 4 g L^−1^, (c) 8 g L^−1^, (d) 12 g L^−1^, (e) 16 g L^−1^ PEG_1000_ and (f) cross-section morphology of the coating P2.

Surface morphologies of MAO coatings formed in different electrolytes and a typical cross-section SEM image of coating P2 are shown in Fig. 3. Compared to the surface of coating P0, the roughness of other coatings is influenced by the level of PEG_1000_. For instance, the smoothness of coating P1 is improved to some extent, and much more significantly for P2, which is smoother with homogenous micro-pore sizes and spatial distribution (Fig. 3(c)). Nonetheless, the roughness tends to rise again with further increasing PEG_1000_. Uniform micro-pores with different diameters and nodules can be seen in Fig. 3(d) while the roughness of coating P4 is close to that of P0 with discontinuous nodules and craters demonstrated in Fig. 3(e). The different morphologies of PEG_1000_-containing coatings result from the alteration of coating growth, which influences the distribution and sizes of micro-pores, nodules and micro-cracks in turn. In addition, as is shown in the cross-section SEM image (Fig. 3(f)), the coating P2 is consist of a porous outer layer, a consecutive transition layer and a dense inner layer with relatively uniform thickness. Above all, there is no apparent discontinuity to the Mg–2Sr substrate in the bond zone, which proves that metallurgical bonding is generated successfully between the ceramic coating and Mg–2Sr alloy.

#### 3.2.2. Surface morphology after SBF immersion

**Fig. 4:**
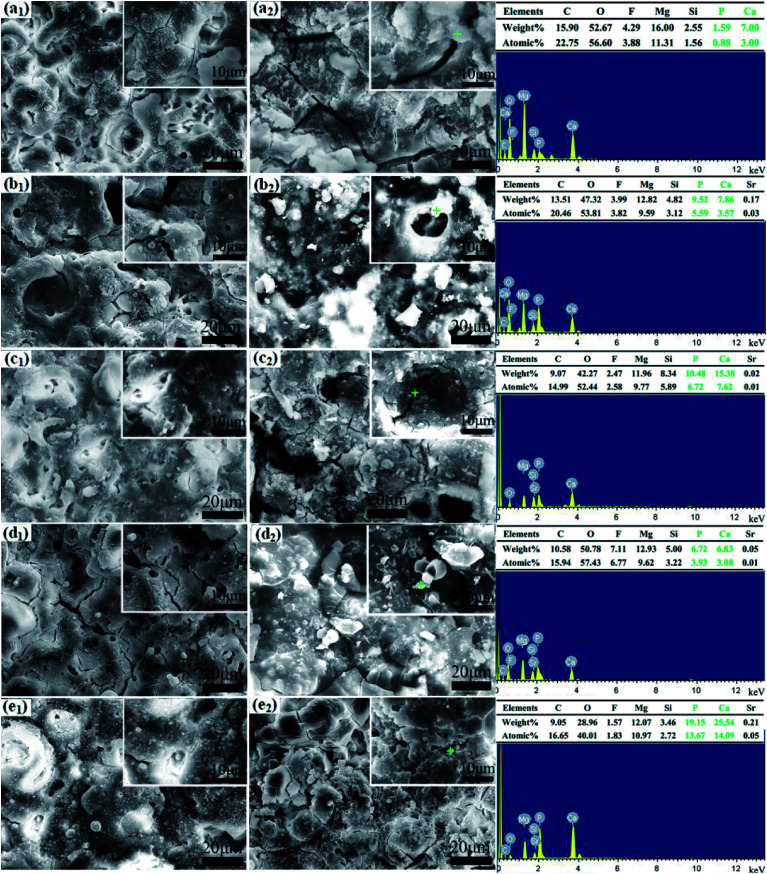
Surface morphologies and elemental compositions of the MAO coatings after immersion in SBF for 1 day and 7 days: the coating with 0 g L^−1^ (a_1_ and a_2_), 4 g L^−1^ (b_1_ and b_2_), 8 g L^−1^ (c_1_ and c_2_), 12 g L^−1^ (d_1_ and d_2_) and 16 g L^−1^ (e_1_ and e_2_) PEG_1000_.

Surface morphologies of MAO coatings after immersing for 1 day and 7 days are compared systematically in Fig. 4. After immersing in SBF solution for 1 day, there is no evident change in the morphology and the typical porous microstructure is still visible along with numerous newly-formed spherical-like particles in various sizes. Granular particles are observed in Fig. 4(a_1_) and (e_1_) while corrosion cracks are found in higher magnification images (Fig. 4(b_1_) and (d_1_)). Coating P2 demonstrated excellent integrity without appreciable defects in this period (Fig. 4(c_1_). New surface morphologies appear after SBF soaking for 7 days. Surfaces of MAO coatings are covered with apatite-like layers different from each other. As for the coating P0 with obvious degradation morphology, the porous microstructure and granular particles disappear while micro-cracks and column apatites are formed (Fig. 4(a_2_)). For coatings P1 and P3 (Fig. 4(b_2_) and (d_2_)), clusters of coral-like and shell-like apatites locate in the open pores and concaves of coatings, respectively. Clusters of spherical and blocky apatites are observed in the micro-cracks on the coating P4 (Fig. 4(e_2_)) where granular and blocky particles with different sizes are generated on the surface with some gathering in corrosion cracks. Moreover, the micro-pores are lager in contrast to those in Fig. 4(c_1_) for the coating P2.

Chemical compositions of MAO coatings after SBF immersion for 7 days are given by EDS analysis. The results indicate that particles from corrosion products are consisted of C, Mg, F, O, Si, Ca and P. A trace of Sr can be detected except for the PEG_1000_-free coating. Interestingly, the contents of Ca and P in spherical and columned particles are higher than those in shell-like particles. In combination with XRD, FT-IR results, SEM morphologies and EDX, these Ca- and P-containing apatites are transformed from calcium phosphates after immersion. Calcium phosphate apatite is the only type of calcium phosphate crystal in calcified tissues such as bones, teeth, calcified cartilage, and cultured matrix produced by osteoblasts,^52,53^ which possesses good biocompatibility and osteoconductivity allowing the formation of bone on its surface.^54,55^ The excellent bioactivity of MAO coatings is proved by the formation of new bone-like apatites after 7 days' immersing.

### 3.3. Thickness, porosity and adhesion

**Fig. 5:**
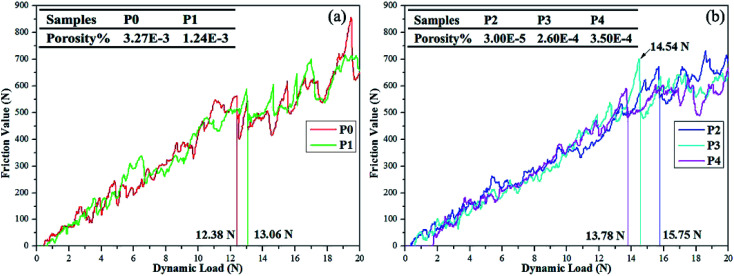
The dynamic loads of (a) coating P0, P1; (b) coating P2, P3, P4.

Measured thicknesses (*X* ± SD, SD refers to the standard deviation) of coatings P0 to P4 are 51.5 ± 2.2, 42.2 ± 2.4, 41.5 ± 2.2, 41.2 ± 1.8 and 39.0 ± 2.0 μm, respectively. Obviously, the thickness of coating P2 is consistent with that observed in the cross-section image in Fig. 3(f). Small differences are found among thicknesses except for the PEG_1000_-free coating with the largest value observed. The hydro soluble PEG_1000_ with certain viscosity may hinder the migration of ions in electrolytes and therefore reduce the ions exchange of the whole system, which can explain the results above reasonably. The coating adhesion can be defined as the dynamic load corresponding to the saltation of friction value. The dynamic loads of P0 to P4 are 12.38 N, 13.06 N, 15.75 N, 14.54 N and 13.78 N, respectively while the porosity is shown in the table embedded in Fig. 5. The dynamic load increases firstly then decreases while the porosity tends to be inverse according to increasing PEG_1000_ contents. Hereby, both the highest adhesion value and lowest porosity are achieved for the coating P2, which implies good adhesion and load-bearing capacity resulting from the metallurgical bonding between the substrate and coating. In a word, porosity is the leading point to dynamic adhesion compared to the thickness, which can be calculated from Stern–Geary equation^56^ and another equation proposed by Liu *et al.*^57^*via* potentiodynamic polarization tests described in the follows:
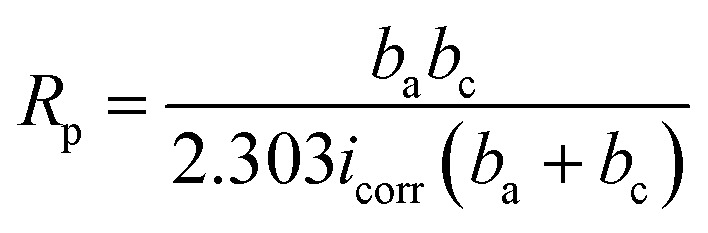

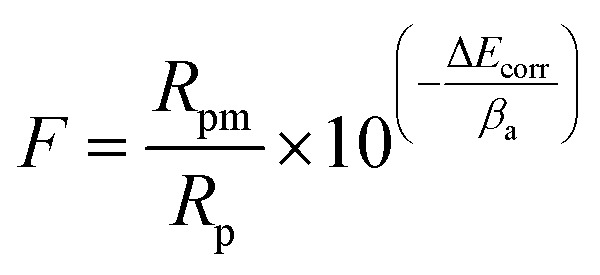
where, *b*_a_ and *b*_c_ are the anodic and cathodic Tafel slopes of the measured samples in the first formula while the *F*, *R*_pm_, *R*_p_, *E*_corr_ and *β*_a_ are the coating porosity, the polarization resistance of the substrate, the polarization resistance of the MAO coated samples, the difference of the corrosion potential between the coated and uncoated samples and the anodic Tafel slope of the uncoated sample, respectively.

**Fig. 6:**
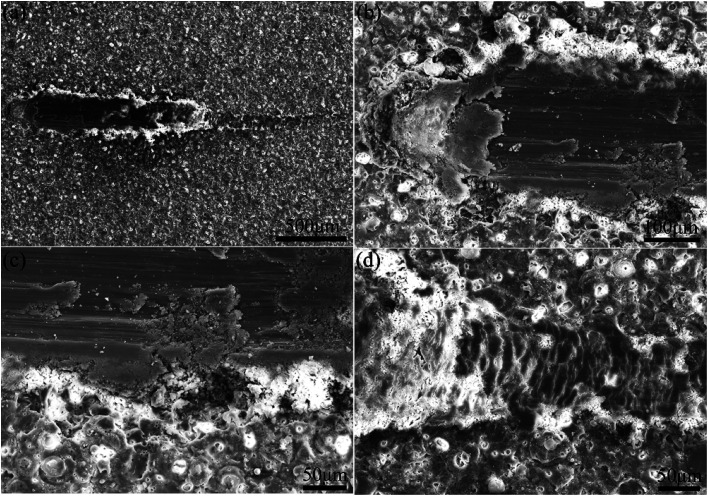
SEM micrographs after the scratch test of coating P2 formed on Mg–2Sr: (a) the whole scratch, (b) the beginning of the scratch, (c) the broken and spalt area of the scratch and (d) the ending of the scratch.

Fig. 6 demonstrates SEM images of scratched coatings prepared in the electrolyte containing 8 g L^−1^ PEG_1000_. According to Fig. 6(a), the external coating is clearly pressed to two sides by the indenter leaving a scratch track as expected. Additionally, Fig. 6(b) shows that the micro-pore structure is indeed apparent at the beginning and then disappears along with the extension of scratch. The coating is penetrated and the substrate is exposed to the air subsequently once saltation of friction value. Stratified spalling phenomenon is visible in the spalt area of the scratch from Fig. 6(c). The terminal of the scratch suggests a scaly structure without micro-pores owing to the movement of indenter as is shown in Fig. 6(d), where the substrate is protected by the coating from partly destruction.

### 3.4. Electrochemical corrosion behaviour and degradability

**Fig. 7:**
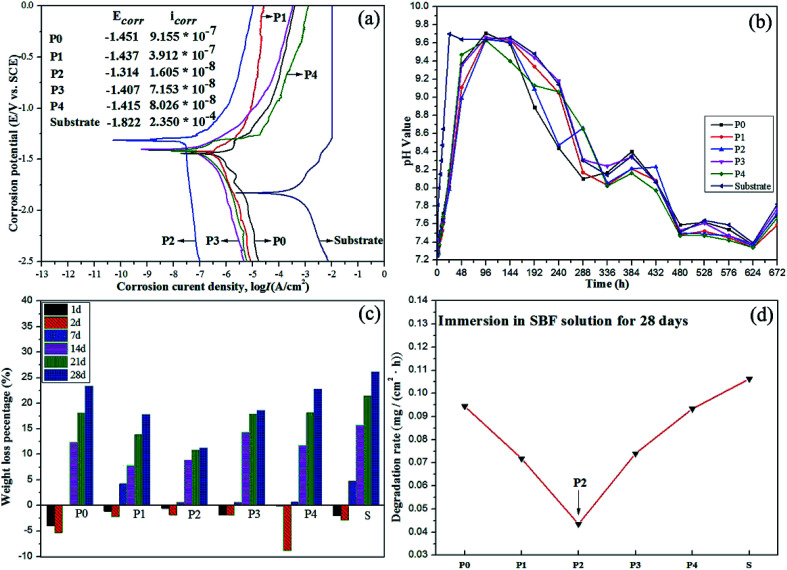
Electrochemical test results of the MAO coatings: (a) polarization curves, (b) pH values, (c) weight loss percentages and (d) degradation rates of substrate and MAO coated samples formed in different electrolytes.

The corrosion resistance and degradability of the MAO coatings can be evaluated by electrochemical polarization curves, pH values, weight loss percentages and degradation rates, as shown in Fig. 7. In particular, corrosion resistance and apatite formation ability are susceptive factors for degradation rates of coatings since the dissolution of samples can be delayed or prevented by formation or growth of apatite-like layers.^1,58^ According to Fig. 7(a), the corrosion potential (*E*_corr_) of MAO coatings shifts positively by 0.37–0.41 V while the corrosion current density (*I*_corr_) is three or four orders of magnitude lower than that of the substrate, which infers that the corrosion resistance of Mg–2Sr modified by the MAO with the introduction of PEG_1000_ is improved to a remarkable degree especially for the coating prepared in the 8 g L^−1^ PEG_1000_-incorporated electrolyte system. The corrosive components are able to invade the micro-pores or micro-cracks easily but fail to penetrate the inner compact layer, which reduces corrosion rates at the end. Fig. 7(b) draws a conclusion that the pH values increase with prolonged exposure to SBF in every 2 days. The line for coating P2 is smoother and reaches the maximum at the sixth day in SBF immersion while that of the substrate demonstrates an obvious fluctuation. After the same exposure time, the pH value of the SBF solution in which the substrate was immersed is relatively higher than those of the SBF solutions in which MAO-coated samples were immersed except for values of the twelfth day. Interestingly, the weight loss percentage of each sample is negative in the first two days and then increases gradually with further soaking as seen in Fig. 7(c), which means that a new layer was formed on the surfaces of samples and then dissolved into the corrosive SBF solution. For the MAO coatings, the expected phases of CaP apatites provides biocompatibility and bioactivity. From the substrate's point of view, weight increase percentages and lower loss percentages in the earlier stage support the implants' stability and mechanical integrity. The weight loss percentage of substrate is higher than those of MAO-coated samples in any exposure period. The highest weight loss percentage of the substrate reaches to 26.10% while coating P2 presents the lowest value of 11.22% after immersion for 28 days. The degradation rates of P0, P1, P2, P3, P4 and the substrate are calculated to 0.09443, 0.0718, 0.04349, 0.074, 0.09334 and 0.10626 mg (cm^2^ h)^−1^ respectively (Fig. 7(d)). MAO-coated samples exhibit much lower degradation rates and better electrochemical corrosion resistance, which implies that MAO coatings are favorable to enhance the corrosion resistance. The incorporation of 8 g L^−1^ PEG_1000_ into the electrolyte provides a further noticeable improvement.

## 4. Discussion

Mg–2Sr alloys undergo a complex process relating to chemical, electrochemical, thermochemical and plasma reaction for MAO coating formation and growth. Mg–2Sr firstly dissolves in electrolyte system and then Mg^2+^, Sr^2+^ are released at the beginning of reaction when a thin layer of oxide film is formed.^59^ Oxygen bubbles on the surface of sample result from oxidation of OH^−^ ions and H_2_O,^60^ which are absorbed on weak parts of the thin layer. Subsequently, the weak layer with the lowest resistance is broken down preferentially accompanied by spark discharge phenomenon. At this moment, coating thickness increases gradually mainly depending on the extensive migration of cations (Mg^2+^, Sr^2+^) and anions (O^2−^, SiO_3_^2−^, PO_4_^2−^) while modest breakdown occurs. Some bubbles are covered by the coatings, in which gases are pulled out because of the high pressure originating in discharge. Therefore, pores are left on the primary oxide coating surface and subsequently become discharge channels of microarc oxidation.^61^ At the end, the oxide film finishes growth process when setting voltage could not breakdown final coating along with sporadic spark appears in the local surface area.

The microstructure, thickness, porosity and adhesive strength can be influenced by introducing nonionic surfactant PEG_1000_. It could adjust the electrolyte activity and then affect the ions exchange to alter the coating growth mechanism. Theoretically, –CH_2_–CH_2_–O– segments of the nonionic surfactant PEG_1000_ are absorbed on the surface of Mg–2Sr alloy with C atoms oriented toward the substrate surface and O atoms oriented toward the solution, which may promote arc discharge and optimise MAO process. With the help of C–C bond to accelerate electron transferring, stronger adhesion can be achieved in the interface between coating and substrate. MAO coating microstructure may also be smoother when modest PEG_1000_ is added in the electrolyte. Nevertheless, excessive –CH_2_–CH_2_–O– segments from hydro soluble PEG_1000_ with certain viscosity may hinder the migration of ions and reduce the electrical conductivity of the whole system to lead gradually reduced film thickness with increasing PEG_1000_ concentrations. Given that the outward migrated cations (Mg^2+^, Sr^2+^) are held back by intensive C atoms absorbed on the substrate and inward migrated anions (O^2−^, SiO_3_^2−^, PO_4_^2−^) fail to close to the cations for the hamper of –CH_2_–CH_2_–O– segments, resulting in loose coating textures with higher porosity and lower adhesion.

Electrochemical corrosion behaviour is influenced by thickness, porosity and cracks of MAO coating. The porous outer layer with micro-pores and micro-cracks cannot hold back the corrosive ions such as Cl^−^, but the dense inner layer is indeed hard to be invaded. Therefore, the electrochemical corrosion property is improved by MAO coatings to great extent because the substrate is free to be corroded by corrosive components, which can be verified by the remarkable low magnitude of corrosion current densities (*I*_corr_) for MAO-coated samples compared to the substrate counterpart. It is necessary to adjust the thickness and microstructure of MAO coatings to enhance electrochemical corrosion resistance by incorporation of effective additives. Lower porosity not the thickness is the leading factor for excellent corrosion resistance for MAO-coated samples because continuously increasing thickness becomes invalid when the microstructure is relatively loose for increasing number of large pores and cracks. Furthermore, there is something associated between adhesion and corrosion resistance due to both of them are linked with porosity. Coatings with stronger adhesive strength tend to possess thicker inner layer and then be harder to be penetrated by corrosive ions, which indicates that improving adhesion is helpful to decrease corrosion current density. In conclusion, the coating P2 possesses the lowest electrochemical corrosion current (1.605 × 10^−8^ A cm^−2^) owing to its minimum porosity, maximum adhesive strength and relative dense successive microstructure.

Degradation of coating is dependent on the period immersing in SBF solution for the balance movement between precipitation of calcium phosphates and dissolution of coating or even substrate. At the beginning stage, small part of dissolution occurs in the corrosive environment resulting from the dissolving of unstable phases such as water-soluble phase TCP. Also, SBF is a metastable calcium phosphate solution supersaturated with respect to bone-like apatites.^62^ Cl^−^ ions in SBF accelerate hydrolysis of CaP phases (Ca_3_(PO_4_)_2_) and SrSiO_3_ to produce Ca^2+^, Sr^2+^, OH^−^, HPO_4_^2−^, and PO_4_^3−^ ions and transformation of MgO and CaO into soluble Mg(Ca)Cl_2_ to increase ions concentration near the coating surface, which is beneficial to ions transferring and precipitation. In the next stage, the micro-pores, micro-cracks and dissolution areas at the beginning stage provide a large number of nucleation sites for HA, Sr-HA and Ca_2_P_2_O_7_. With further ions releasing and more nucleation sites valid, nucleation of HA, Sr-HA, Ca_2_P_2_O_7_ and other calcium phosphates is induced around the solution-coating interface, after which the nuclei spontaneously grow by quickly consuming Ca^2+^, Sr^2+^ and HPO_4_^2−^ from metastable supersaturated SBF solution. In the present work, the formation and growth of these calcium phosphates on samples' surface outweigh weight losses from coating dissolving especially for the immersion in the first two days. During the third stage, serious dissolution of coating damages the balance because the corrosive Cl^−^ ions may penetrate the dissolved coating into the substrate. SBF solution can be directly contacted with Mg–2Sr alloy and then intensive corrosion begins, from which we can draw that the precipitating ability of calcium phosphates is limited to the corrosion resistance of the coating. As a result, weight lose percentages of coatings increase with longer immersion time.

## 5. Conclusion

Corrosion behaviours of MAO coatings prepared on Mg–2Sr alloy are influenced after inducing PEG_1000_ to electrolyte systems. The following conclusions can be drawn:

(1) Biocompatible MAO coatings are formed successfully in five different electrolytes with similar thicknesses except for a thicker one formed in PEG_1000_-free electrolyte, which are mainly composed of Mg, MgO, CaO, SrSiO_3_, Sr_2_Mg_17_ and Ca_3_(PO_4_)_2_ regardless of the PEG_1000_ concentrations. Adhesive strength of the coatings is improved by the addition of PEG_1000_ to different degrees and the maximum value is obtained when 8 g L^−1^ PEG_1000_ is incorporated.

(2) Corrosion resistance behaviours and degradability are modified especially by PEG_1000_-incorporated MAO coatings due to its solubility and viscidity. The segments –CH_2_–CH_2_–O– of the nonionic surfactant PEG_1000_ are absorbed on the surface of Mg–2Sr alloy to promote arc discharge and ions exchange. The highest corrosion resistance and lowest degradation rate are also observed in the 8 g L^−1^ PEG_1000_-incorporated coatings.

(3) A new layer containing CaP apatites are formed on the surfaces of MAO coatings after soaking in the SBF solution, which is beneficial to improve the biocompatibility and bioactivity. Weight increase in the first two days and lower loss percentages support the implants' stability and mechanical integrity, which is essential to the healing and growth of the surrounding tissues. Altogether, the coating P2 exhibits lower porosity, higher adhesive strength, better apatite-inducing ability and slower degradation rate.

## Conflicts of interest

There are no conflicts to declare.

## Supplementary Material
